# Pharmacoeconomics; an Appropriate Tool for Policy Makers or Just a New Field of Research in Iran? 

**Published:** 2012

**Authors:** Majid Davari

**Affiliations:** Health Management and Economics Research Center, Isfahan University of Medical Sciences, Isfahan, Iran.

Pharmacoeconomics is defined as the science of identifying and comparing costs and the consequences of drug therapy in health care programs. As a sub-discipline of health economics, pharmacoeconomics, is fairly a new research discipline in the world which has come about mainly in response to economic pressures on health systems. The root of this pressure lies in the mismatch of enhanced demand for health care and pharmaceutical services on one hand, and limitations on available resources on the other.

    Iranian health system has also faced difficulties in managing its limited resources and containment of the health and pharmaceutical expenses. Pharmaceutical expenditures in Iran have increased from 353 Million US $ in 2001 to 2.45 Billion US $ in 2009. This is while the whole number of pharmaceutical sales has increased just from 18.6 Billion to 28.5 Billion during the same period. The figures show that the growth rate of the pharmaceutical expenditures is nearly 11 times greater than the growth rate in the number of pharmaceutical sales.

   It has been suggested that innovation and advances in new and high cost pharmaceuticals, appearance of new diseases, and enhancing patients’ expectations are of the main causes of a sharp increase in pharmaceutical expenditures worldwide. But, in addition to these common causes accounting for the quick increase in health and pharmaceutical expenditures worldwide, there are some specific reasons for Iran which could explain why this trend has happened to the Iranian pharmaceutical expenditures so sharply.

   Introduction of market-oriented reform (even though imperfectly), including privatization of the state-owned pharmaceutical manufactures, reduction of medicine subsidies, and allowing easier access to foreign markets could be considered as important elements, which have affected the total pharmaceutical expenditures considerably.

    For whatever reason, which is important enough to be discussed in more details elsewhere, it is obvious that the health system is faced with the challenge of a sharp increase in pharmaceutical expenditures. As one of the consequences of this increase, health insurance organizations have reduced their pharmaceutical coverage significantly. The result of this problem has appeared in patient out of pocket spending for pharmaceuticals, which is increased to around 65 percent. This could be an important warning sign, which clearly shows that the health insurance organizations are not able to manage the trend of pharmaceutical expenditures in the country.

    It is reasonable that there will never be sufficient resources to achieve all ideal objectives in health care, even for rich countries. But, at the same time, it is the responsibility of health policy makers to manage and optimize the use of limited resources effectively.

   Pharmacoeconomics could help pharmaceutical policy makers to optimize the allocation of their resources successfully. It could help to provide a better understanding of the value of medicines through analysis of their costs, as well as their consequences. Using the results of Pharmacoeconomics, policy makers can understand the value added to the society by using any pharmaceutical. This can help them to recognize which treatment may be the most efficient strategy for treating patients. Using the efficient treatment strategies could, in turn, increase the efficiency of the pharmaceutical services. But, as it is important to use pharmaceuticals efficiently, it is also essential to apply Pharmacoeconomics in an appropriate way to be most effective. One of the key elements in this regard is to provide an appropriate bridge between Pharmacoeconomics too as a science and Pharmacoeconomics as a policy and management tool. Pharmacoeconomics as a science focuses on the methodological points,which should be considered in order to have a valid and reliable analysis. It could focus on subjects such as: points to be considered in measuring effectiveness, pitfalls in the inclusion of costs, the reasons and the size of discounts in economic evaluations and so on. Pharmacoeconomics as a policy and management tool should concern more about selecting topics, persons or organizations which should perform economic evaluations; organizations which should be taken for the responsibility of applying the results of Pharmacoeconomics studies, methods for applying its results effectively, the level of cost effectiveness threshold, scale of fairness and acceptability, and so forth.

   Requesting for Pharmacoeconomics the studies in Iran is being increased in recent years; mainly in response to the shortage of financial resources. It is clear that Pharmacoeconomics is going to play a more important role in pharmaceutical policy in the country in near future. While this could be considered as a step forward to the application of Pharmacoeconomics in pharmaceutical system, pharmaceutical authorities need to clarify the essential factors which could have an effective and acceptable role in Pharmacoeconomics studies in the pharmaceutical system.

    At the time being, food and drug organisation, health department, and health insurance organizations are asking for economic evaluation analysis separately and without any systematic cooperation. This could increase inefficiency itself. Establishment of a systematic connection between various departments, which are customer of economic evaluation studies, is an important strategy to avoid duplication and unnecessary expenses.

   Running economic evaluations is not costless itself. It is important to clarify who is responsible for the provision of financial support for such studies; and who would be benefited more from implementation of economic evaluation results in the Pharmaceutical system.

   The last but not least point to consider is that, although the application of Pharmacoeconomics in designing policy may help to increase the efficiency of the system, it does not necessarily mean that the result would be a reduction in pharmaceutical expenditures. On the contrary, the results in some countries, like England and Wales, have shown that their pharmaceutical expenditures have also increased after development of National Institute for Health and Clinical Excellence (NICE) in 1999. Nevertheless, this may result in increasing the efficiency of the healthcare services, beside the provision of better health care services to the population. This reality could help us to understand that Pharmacoeconomics is not the answer of all challenges and issues in pharmaceutical policy. It could help decision makers to use their limited resources more efficiently if, and only if, it is brought about to practice appropriately.

    In summary, Pharmacoeconomics in Iran, and also in other countries, could help policy makers to better design and drive their policy objectives more efficiently. But it is necessary to clarify, in advance, the expectations from applying Pharmacoeconomics, department to be in charge of applying the results of Pharmacoeconomics studies, values and standards to be considered in doing such studies, financial resources for performing and implementing its results, and its potential impact on various parts of pharmaceutical system including patients, pharmaceutical industry, and health insurance organizations. Clariflying these point will assist pharmaceutical policy makers to benefit from pharmacoeconomic more effectively.

*Majid Davari, is currently working at the Health Management and Economics Research Center, Isfahan University of Medical Sciences, Isfahan, Iran. at the following e-mail address: davari@pharm.mui.ac.ir*

